# Controversial Aspects of Diagnostics and Therapy of Idiopathic Condylar Resorption: An Analysis of Evidence- and Consensus-Based Recommendations Based on an Interdisciplinary Guideline Project

**DOI:** 10.3390/jcm12154946

**Published:** 2023-07-27

**Authors:** Merle Riechmann, Christopher Schmidt, M. Oliver Ahlers, Ima Feurer, Johannes Kleinheinz, Andreas Kolk, Christoph Pautke, Andreas Schön, Marcus Teschke, Astrid Toferer, Christopher J. Lux, Christian Kirschneck, Gabriele A. Krombach, Peter Ottl, Ulla Vieth, Johanna Stengel, Caroline Völker, Andreas Neff

**Affiliations:** 1 Department of Oral and Craniomaxillofacial Surgery, University Hospital Gießen and Marburg GmbH, University Hospital Marburg, and Faculty of Medicine, Philipps University, 35043 Marburg, Germany; merlee.riechmann@t-online.de (M.R.); schmidt-cb@web.de (C.S.); johanna.99.stengel@gmail.com (J.S.); voelkerc@students.uni-marburg.de (C.V.); 2 Department for Radiology and Nuclear Medicine, GPR Hospital Rüsselsheim, 65428 Rüsselsheim am Main, Germany; 3 Medical Practice, CMD-Centrum Hamburg-Eppendorf, 20251 Hamburg, Germany; oliver.ahlers@cmd-centrum.de; 4 Department of Prosthetic Dentistry, Center for Dental and Oral Medicine, University Medical Center, Hamburg-Eppendorf, 20246 Hamburg, Germany; 5 Physiotherapeutic Practice & Orthopedic Manual Therapy, 78315 Radolfzell-Böhringen, Germany; ima.feurer@t-online.de; 6 Department of Craniomaxillofacial Surgery, University Hospital Münster, Westfälische Wilhelms-University Münster, 48149 Münster, Germany; johannes.kleinheinz@ukmuenster.de; 7 Department of Oral and Maxillofacial Surgery, Medical University of Innsbruck, 6020 Innsbruck, Austria; andreas.kolk@i-med.ac.at; 8 Medical Practice & Clinic for Oral and Craniomaxillofacial Surgery, 80333 München, Germany; christoph.pautke@gmx.net; 9 Department of Oral and Craniomaxillofacial Surgery, University Hospital Bonn, Rheinische Friedrich-Wilhelms-University, 53127 Bonn, Germany; andreas.schoen@mkg-troisdorf.de; 10 Medical Practice for Oral and Craniomaxillofacial Surgery, 28195 Bremen, Germany; marcus.teschke@me.com; 11 Medical Practice for Oral and Craniomaxillofacial Surgery, 8301 Laßnitzhöhe, Austria; astrid.toferer@gmx.de; 12 Polyclinic for Orthodontics, University Hospital Heidelberg, 69120 Heidelberg, Germany; christopher.lux@med.uni-heidelberg.de; 13 Polyclinic for Orthodontics, University Hospital Regensburg, 93053 Regensburg, Germany; christian.kirschneck@ukr.de; 14 Department of Diagnostic and Interventional Radiology, University Hospital Giessen, 35392 Giessen, Germany; gabriele.krombach@uniklinikum-giessen.de; 15 Department of Prosthodontics and Materials Sciences, Rostock University Medical Center, 18057 Rostock, Germany; peter.ottl@uni-rostock.de; 16 Department of Life, Light and Matter, University of Rostock, 18059 Rostock, Germany; 17 Medical Practice for General Medicine, 36088 Hünfeld, Germany; ullaprechel@gmx.de

**Keywords:** idiopathic condylar resorption, condylar resorptions, temporomandibular joint, guideline, Delphi method, consensus

## Abstract

Idiopathic condylar resorption (ICR), though a rare event, is associated with severe detrimental sequelae for the patient. To date, the etiology remains unknown, and treatment strategies are highly controversial. Therefore, the aim of this study is to present an analysis of the consensus- and evidence-based approach to ICR by a German interdisciplinary guideline project of the AWMF (Association of the Scientific Medical Societies in Germany). Following a systematic literature search, including 56 (out of an initial 97) publications, with a predominantly low level of evidence (LoE), two independent working groups (oral and maxillofacial surgery and interdisciplinary, respectively) voted on a draft comprising 25 recommendations in a standardized anonymized and blinded Delphi procedure. While the results of the votes were relatively homogeneous, the interdisciplinary phase required a significantly higher number of rounds (*p* < 0.001). Most of the controversial recommendations were related to initial imaging (with consensus on CT/CBCT as the current diagnostic standard for imaging), pharmacotherapy (no recommendation due to lack of evidence), discopexy (no recommendation possible due to low LoE) and timing of orthognathic surgery (with consensus on two-staged procedures after invasive TMJ surgery, except for single-stage procedures if combined with total joint reconstruction). Overall, the Delphi procedure resulted in an interdisciplinary guideline offering the best possible evidence- and consensus-based expertise to date in the diagnosis and treatment of ICR.

## 1. Introduction

Idiopathic condylar resorption (ICR) is a very rare clinical entity that should be treated as a diagnosis of exclusion. While the resorptions observed can be attributed predominantly to a secondary genesis, these secondary condylar resorptions are usually also subsumed under the term “ICR” in clinical usage. Idiopathic condylar resorption is characterized by osteolysis of the mandibular condylar process. According to our literature search, the resorption process has never been described to progress deeper than the superior aspect of the sigmoid notch, i.e., the border between ramus and condylar process [1,2,3].

A differentiation is made between active (progressive) and stable (non-progressive) resorption. The disease usually affects both temporomandibular joints [1,4]; however, an asymmetrical course is also possible [5,6]. The dentofacial manifestations of ICR overlap considerably with degenerative joint diseases, especially with osteoarthritis and juvenile idiopathic arthritis (JIA) of the TMJ [2,3]. Since both entities progress in a completely different way and require different therapeutic consequences, early differential diagnosis is crucial [2].

ICR is a pathology observed mainly in women between the ages of 15 and 35 years [1,5,6,7,8,9,10,11]. The reported incidence in association with maxillofacial surgeries ranges between 1% and as much as 31% [12]. According to the international, especially Anglo-American, literature, mainly orthognathic surgeries are mentioned as etiologically relevant factors [5,12,13]. While the high incidence rates mentioned above do not correspond with the experience under the standard surgical procedures applied, e.g., in German-speaking countries, they do lead to a certain degree of uncertainty among patients and practitioners.

In most cases of condylar resorption, the causes remain unknown, and different approaches to aetiology and pathogenesis result in differing views regarding the therapeutic approach to be adopted. Conflicting study results complicate consensus finding [8,14,15,16].

Due to the comparatively low incidence, the current data on idiopathic condylar resorption are limited [11]. Existing studies are mostly retrospective case studies with small case numbers and short follow-up [5,7,10,17,18,19]. The studies available to date with a higher level of evidence (LoE 3 and higher) are mainly diagnostic studies without statements regarding methods of treatment [2,14].

Considering the uncertainty among patients and practitioners resulting from the epidemiologically poor data, sufficient evidence and standardized approaches are still needed in many areas of clinical management, hence the great significance of the interdisciplinary, evidence- and consensus-based guideline published online in Germany by the Association of the Scientific Medical Societies in Germany (Arbeitsgemeinschaft der Wissenschaftlichen Medizinischen Fachgesellschaften—AWMF) as an evidence- and consensus-based S3 guideline in 03/2023. The aim of this study is to identify and analyse the issues subject to controversial discussion during the development of the above S3 guideline, based on a systematic literature search and an interdisciplinary expert consensus of the mandated representatives of the contributing scientific societies.

## 2. Materials und Methods

### 2.1. Systematic Literature Search

The foundation for the current guideline was the previous S3 guideline on ICR (06/2016), which itself represents a comprehensively updated and revised version of the preceding S1 guideline (11/2009) on ICR.

Initially, a search for national and international guidelines was conducted within databases, i.e., PubMed, Cochrane, AWMF, www.guideline.gov (access date 16 June 2021) and www.nice.org.uk (access date 16 June 2021). However, no relevant guidelines addressing ICR could be identified.

Both the initial and subsequent literature searches were conducted using the syntax “idiopathic condylar resorption [AND] temporomandibular joint”. The guideline working group decided against limiting the search to specific years of publication, study types or research questions due to the limited availability of relevant studies. The inclusion criterion was publication in the German or English language. The specific research questions resulting from the initial search were further explored using the PICOTS scheme.

**Figure 1:**
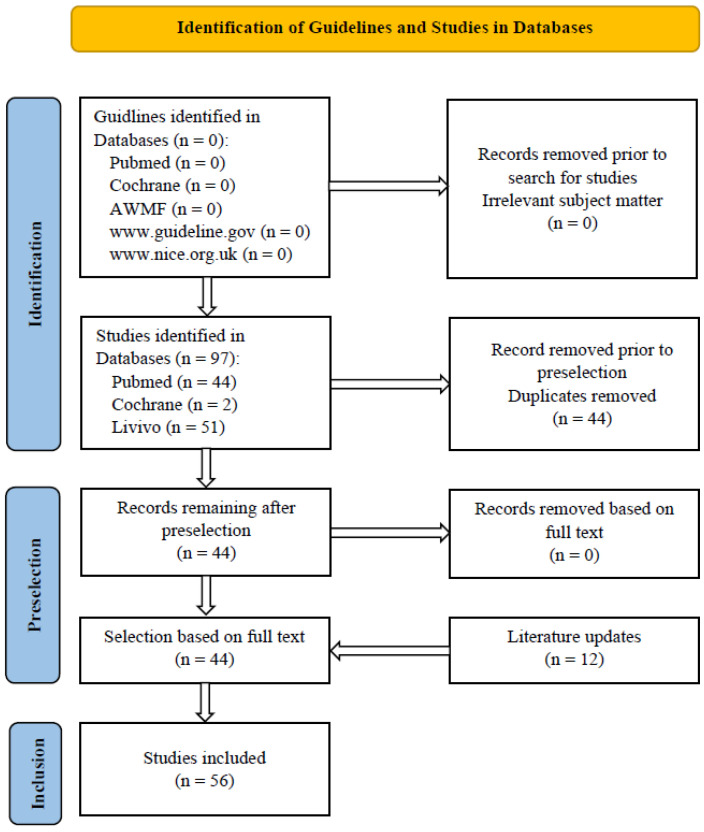
Literature search—2021/2022 flow chart (last updated 12/2022).

In addition to the 63 sources from the previous S3 guideline (06/2016), a further 47 sources were included in the guideline as part of the 03/2023 update. Titles and abstracts of potentially eligible records were reviewed and assessed by two medical experts independently (M.R. and C.V.), and duplicates and studies not relevant for the guideline were removed. A further selection was made from the remaining publications based on a review of their full text. Additional sources were included in the guideline after a manual search and following an update (Figure 1). In cases of disagreement between the experts, a third expert was consulted (A.N.).

### 2.2. Assessment of Evidence

The level of evidence (LoE) was assessed by two medical experts (M.R. and C.V.) independently, based on the Oxford Criteria 2011 (Table 1). In case of doubt, a third expert was consulted (A.N.).

The methodological quality was assessed in compliance with the SIGN Checklists (https://www.sign.ac.uk/what-we-do/methodology/checklists/; access date 29 June 2021) by two experts independently (M.R. and C.V.) and classified into four categories (Table 2). In cases of essential disagreement between the experts, a third expert was consulted (A.N.).

With SIGN Checklists available as tools for assessment of methodological quality for levels of evidence 1 to 3 only, and due to the widely heterogenous quality of studies of levels of evidence 4 and 5, an additional assessment of clinical relevance was introduced and applied to all study types and levels of evidence. Depending on the study subject and methodology, the criteria applied were as follows: relevance of research question and target numbers; patient sample size, inclusion and exclusion criteria; disclosure of patient characteristics; duration of follow-up; “lost-to-follow-up” rates; and suitability for comparison of intervention and control group. Assessments were made in a similar manner to SIGN Checklists by two independent experts (M.R. and C.V.) as outlined in Table 3. In case of doubt, a third expert was consulted (A.N.).

### 2.3. Wording of Recommendation and Structured Consensus Procedure

Based on the systematic literature search, and following the identical methodology as in previous AWMF guidelines [20,21], an initial draft guideline was compiled by the steering group (M.R. and A.N.) and distributed by e-mail. The subsequent consensus process had two stages. In the first phase (Consensus Phase Oral and Maxillofacial Surgery/OMFS (K1)), the draft guideline was agreed on through a Delphi process by the DGMKG (Deutsche Gesellschaft für Mund-, Kiefer- und Gesichtschirurgie) TMJ surgery working group. Each member of the panel had one vote in this phase. The results of this initial consensus phase (OMFS (K1)) were further amended and modified in the second phase (interdisciplinary consensus phase (K2)) by mandated representatives of the participating specialist medical societies. Analogous to the first phase, each participating specialist society had one vote in the interdisciplinary vote K2.

The structured consensus was arrived at by means of a Delphi process (Muche-Borowski C, Selbmann HK, Nothacker M, Müller W, Kopp I: AWMF-Regelwerk “Leitlinien”. http://www.awmf.org/leitlinien/awmf-regelwerk.html (accessed on 1 September 2022)) implemented by e-mail correspondence, which offered the additional advantage of an anonymized and blinded vote. During this process, participants had the options of assigning “shall”, “should” or “may” for “Strength of Recommendation” (equivalent to grade of recommendation A, B and 0 respectively) and of abstention. Furthermore, participants had the opportunity to submit questions, and comments and propose text alterations. As were the results of the vote, these were presented anonymized in the subsequent rounds and voted on, if applicable. The results were assessed by an independent nonvoting member of the steering group (M.R.) (Table 4).

**Figure 2:**
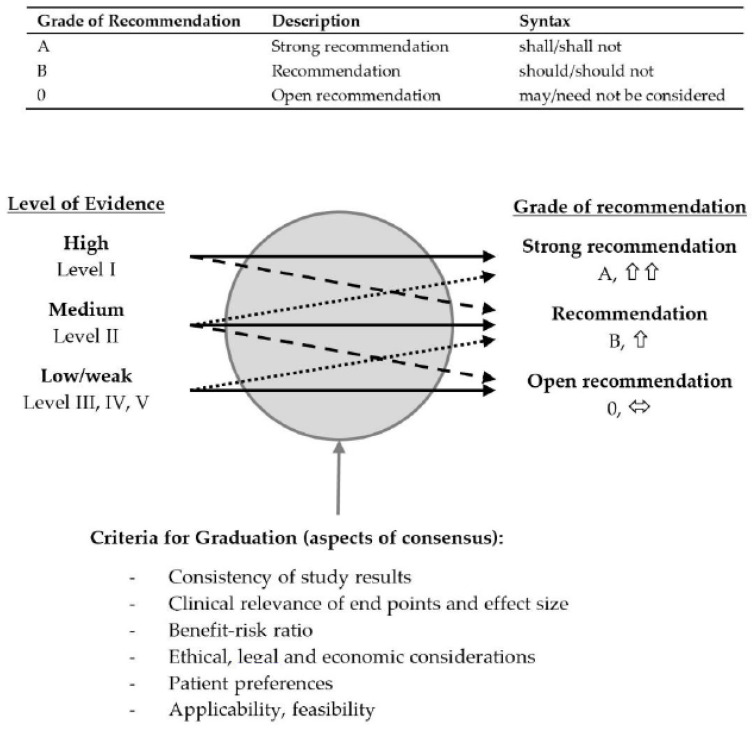
Classification of grades of recommendations according to AWMF rules and standards.

Strength of recommendation (grade) was determined based mainly on the level of evidence but also took into consideration other criteria such as ethical, legal and economic aspects together with clinical experience, feasibility in everyday practice, benefit–risk analysis for those affected, and suitability for the patient target group and for the German health system (Figure 2). Recommendations which could not be sufficiently supported by references in the literature within the meaning of “good clinical practice” (grade of recommendation A with evidence level 4 or 5) were rated as “Expert Consensus”. The Discussion section of this publication states the level of evidence (1–5), grade of recommendation (A: strong recommendation, B: recommendation, 0: open recommendation, EC: expert consensus) and strength of consensus (↑↑: strong consensus, ↑ consensus) for each recommendation. For statements, both evidence level and strength of consensus are provided.

The final version was consented to in an interdisciplinary fashion by all the representatives of the participating specialist societies as well as by the DGMKG. After a structured consensus was arrived at by Delphi process, approbation of the guideline by the board of directors of the participating scientific societies followed and it was published online by the AWMF on 3 April 2023 (https://register.awmf.org/de/leitlinien/detail/007-066, access date 3 April 2023).

### 2.4. Statistics

A comparison was performed between the two consensus phases (initial consensus phase OMFS (K1) vs. interdisciplinary consensus phase (K2)) regarding the variables “consensus” (in percent), “number of rounds”, “strength of consensus” (not approved by majority, approved by majority, consensus and strong consensus) and “proportion of abstentions” (in percent). As “proportion of abstentions” remained unchanged between draft stage (K1) and interdisciplinary consensus (K2), this variable was dismissed.

For the purpose of calculations for the variables “number of rounds”, “consensus” and “strength of consensus”, the Mann–Whitney test and the Fisher’s exact test were applied, respectively. The level of significance was defined as *p* < 0.05.

## 3. Results

### 3.1. Systematic Literature Research

The initial search for national and international guidelines conducted in the databases PubMed, Cochrane, AWMF, www.guideline.gov (access date 16 June 2021) and www.nice.org.uk (access date 16 June 2021) did not produce any relevant guidelines addressing ICR.

The further literature search regarding publications on idiopathic condylar resorption returned a total of 97 records. After selection by title, abstract and full text, then after hand search and additional updates to the literature, a total of 56 sources were included in the guideline (for details, see Figure 1: literature search—2021/2022 flow chart). The initial literature search was performed in May 2021; the sources were again updated in August 2022, with a final update before submission to the AWMF in February 2023. Due to the limited evidence base (no/only few systematic studies in the literature of grades 1, 2 and 3, primary sources, especially evidence levels 4 and 5), case-control studies with evidence level 4 (e.g., case series) and 5 (case reports) were also included in the guideline [21,22].

### 3.2. Consensus Phase

#### 3.2.1. OMFS Consensus Phase (K1) (Initial Draft Version Consensus)

Seven members of the guideline group “TMJS” of the German Association for Oral and Maxillofacial Surgery (Deutsche Gesellschaft für Mund-, Kiefer- und Gesichtschirurgie—DGMKG) were requested to participate in the guideline project on idiopathic condylar resorption. All seven members agreed to participate in the specific working group in the initial consensus phase (see Supplementary Table S1). The initial consensus phase (K1) lasted from January 2022 to June 2022 and included four Delphi rounds, in which 24 graded recommendations were voted on.

#### 3.2.2. Interdisciplinary Consensus Phase (K2)

Furthermore, twelve scientific societies and four patient associations were requested to participate in the interdisciplinary consensus phase. In total, five scientific societies and no patient association agreed to participate (see Supplementary Table S2). The interdisciplinary consensus phase lasted from August 2022 to December 2022 and included two Delphi rounds, in which 25 graded recommendations were voted on. The structured consensus process and approbation of the guideline by the board of directors of the participating scientific societies was completed in February 2023.

#### 3.2.3. Statistical Analysis of the Consensus Process

In the initial OMFS consensus phase (K1), 23/24 (95.8%) of the recommendations achieved a “strong consensus” and 1/24 (4.2%) a “consensus”. In the interdisciplinary consensus phase, 23/25 (92.0%) of the recommendations resulted in a “strong consensus” and 2/25 (8.0%) in a “consensus”.

There were no statistically significant differences in the consensus percentages or the strength of consensus between the initial consensus phase OMFS (K1) and the interdisciplinary consensus phase (K2) (see Table 5, Table 6 and Table 7).

In the initial OMFS consensus phase (K1), 11/22 (50.0%) of recommendations required a second voting round. In addition, two further recommendations were submitted in the third voting round, which also required a further round of voting. In the interdisciplinary consensus phase (K2), 9/25 (36.0%) of the recommendations required a second voting round.

There was a statistically significant difference in the number of rounds needed (*p* < 0.001) (see Table 8, Table 9 and Table 10).

Consensus in percent is shown in the Table 5 and Table 6.

**Table 5:** Consensus in percent in the OMFS consensus (K1) and the interdisciplinary consensus (K2), respectively.

			Frequency	Percent	Valid Percent	Cumulative Percent
K1	Valid	0.86	1	4.0	4.2	4.2
		1.00	23	92.0	95.8	100.0
		Total	23	96.0	100.0	
K2	Valid	0.83	2	8.0	8.0	8.0
		1.00	23	92.0	92.0	100.0
		Total	25	100.0	100.0	

**Table 6:** Test statistics of consensus in percent.

	OMFS Consensus (K1)	Interdisciplinary Consensus (K2)	Total
Mean	0.9940	0.9867	0.9903
N	24	25	49
Std. deviation	0.02916	0.04615	0.03855
Median	1.0000	1.0000	1.0000
Mann–Whitney U (two-tailed): 0.547			

Strength of consensus is shown in Table 7.

**Table 7:** Test statistics of strength of consensus.

			Consensus	Strong Consensus	Total
Option	K1	Count	1	23	24
		% within option	4.2%	95.8%	100.0%
	K2	Count	2	23	25
		% within option	8.0%	92.0%	100.0%
Total		Count	3	46	49
		% within option	6.1%	93.9%	100.0%
Fisher’s exact test: 1.000					

Number of rounds is shown in Table 8, Table 9 and Table 10.

**Table 8:** Number of rounds of the initial OMFS consensus phase (K1).

Valid		Frequency	Percent	Valid Percent	Cumulative Percent
	1.00	1	4.0	4.0	4.0
	2.00	22	88.0	88.0	92.0
	3.00	2	8.0	8.0	100.0
	Total	25	100.0	100.0	

**Table 9:** Number of rounds of the interdisciplinary consensus phase (K2).

Valid		Frequency	Percent	Valid Percent	Cumulative Percent
	1.00	16	64.0	64.0	64.0
	2.00	29	36.0	36.0	100.0
	Total	25	100.0	100.0	

**Table 10:** Test statistics of number of rounds.

	Number of Rounds K1	Number of Rounds K2
Mean	2.0400	1.3600
Median	2.0000	1.0000
Std. deviation	0.35119	0.48990
Mann–Whitney U (two-tailed): <0.001		

#### 3.2.4. Identification of Controversial Areas in the Consensus Process

There was a need for prolonged discussion on certain recommendations and topics, resulting in a prolonged consensus process during the Delphi procedure. A voting result was defined as controversial according to the below criteria:

- Criterion 1: consensus not achieved (agreement <75%) in at least one round.
- Criterion 2: modification of text required to achieve a higher level of consensus (from “not approved by majority” (≤50%) to “approved by majority” (51–75%) or from “consensus” (76–95%) to “strong consensus” (>95%)).

According to these criteria, 9 out of 25 of the recommendations (equivalent to 36.0%) could be identified as controversial in the Delphi procedure. Out of the total of same nine recommendations, consensus could not be achieved (agreement < 75%) in at least one round for seven recommendations, and a modification of the text was required to achieve a higher level of consensus in three recommendations. One of these, nine recommendations met both criteria.

In terms of content, these controversial recommendations involved the following topics (for details, see Table 11):

- Diagnostics: three-dimensional imaging (CT/CBCT) for further diagnostics; three-dimensional imaging (CT/CBCT) to document initial presentation and disease progression; CT or CBCT for specific questions regarding bony structures; MRI scan for specific questions regarding soft tissue; and contrast-enhanced MRI scan to rule out or confirm an autoimmune or rheumatic disease as the cause of the ICR.
- Therapy: in cases of failure of conservative therapy, condylectomy with subsequent reconstruction; arthroplasty as a two-stage procedure, when required in combination with orthognathic surgery—in such cases, arthroplasty procedure first, followed by orthognathic surgery; total alloplastic joint replacement if adequate conservative and surgical measures prove unsuccessful; and reconstructive procedures in a single-stage approach if a combination with orthognathic surgical realignment is required.

**Table 11:** Controversial recommendations. Legend: Grade: grade of recommendation A (strong recommendation)/B (recommendation)/0 (recommendation open); LoE: level of evidence; K1: Initial Consensus Phase OMFS; and K2: Interdisciplinary Consensus.

Item (Final Version)	LoE	Grade	Criteria	Comment/Discussion	Adaptation
1. Three-dimensional imaging (CT/CBCT) shall be applied for further diagnosis and treatment planning or to rule out differential diagnosis.	4/k++	A	Criterion 1: K1.1 Approved by majority (57%) K2.1 Approved by majority (66%)		Not adapted due to missing comments/discussion and insufficient evidence
2. Three-dimensional imaging (CT/CBCT) is the current standard for imaging and documenting the extent of disease and ruling out other differential diagnoses at initial presentation, thus it should be used to document initial presentation and disease progression.	4/k++	B	Criterion 2: K2.1 Consensus (83%) → Adaptation of text	After consultation with the competent scientific association on questions concerning imaging and request for expert assessment modification of text in accordance with the state of art	Modification of text
3. For specific questions regarding bony structures, CT or CBCT should be used as a diagnostic tool.	4/k++	B	Criterion 2: K1.1 Consensus (86%) → Adaptation of text	The examination using cbct was assessed as equivalent to the examination using CT, and therefore supplemented in a text adaptation	Modification of text
4. An MRI scan can provide important additional information for the choice of surgical treatment and for clarification of differential diagnoses, especially for evaluation of soft tissue, especially the disc.	4/k+	0	Criterion 1: K1.1 Approved by majority (57%)		Not adapted due to missing comments/discussion and insufficient evidence
5. In order to exclude or further verify an autoimmune or rheumatic disease as the cause, primarily contrast-enhanced MR diagnostics of the temporomandibular joint should be performed, serological diagnosis only if the result is unclear.	4/k+	B	Criterion 1: K1.1 Not approved by majority (43%)	According to the current German S3 guideline “Inflammatory diseases of the temporomandibular joint—Juvenile idiopathic arthritis and rheumatoid arthritis of the temporomandibular joint”, contrast-enhanced MR diagnostics of the temporomandibular joint are primarily indicated to verify juvenile idiopathic arthritis or rheumatoid arthritis of the temporomandibular joint.	Based on the high level of evidence available (S3 guideline) and subsequent discussion, the initially dissenting guideline group members later agreed with the majority opinion
6. If it is not possible to sufficiently control the symptoms of active condylar resorption (pain, functional limitations) by conservative measures, condylectomy with subsequent reconstruction may be indicated, e.g., from rib cartilage (CCG), or comparable autologous procedures, or use of microsurgical grafts, or total alloplastic joint replacement (cf. S3 Guideline No. 007/106 “Total alloplastic temporomandibular joint replacement”, status 04/2020), if necessary in combination with orthognathic surgery.	4/k+	0	Criterion 1 and 2: K1.1 Approved by majority (71%) → Adaptation of text	Option to perform reconstruction after condylectomy with microsurgical grafts was added	Modification of text
7. Arthroplastic procedures, e.g., for disc repositioning, condylar shave or similar, should generally be performed as a two-stage procedure, if required in combination with orthognathic surgery. The arthroplasty procedure should be performed first, followed by orthognathic surgery.	EC	B	Criterion 1: K1.3 Not approved by majority (43%)		Not adaptated due to missing comments/discussion and insuffi-cient evidence
8. If adequate conservative and surgical interventions with autologous reconstruction prove unsuccessful, or after multiple operations performed in the region, the indication for arthroplasty with total alloplastic joint replacement should be considered, if symptoms are sufficiently severe.	4/k+	B	Criterion 1: K1.1 Not approved by majority (43%)	The previous recommendation contradicts the recommendation of the current S3 guideline “Total Alloplastic Jaw Joint Replacement”, rendering the previous recommendation obsolete	Based on the high level of evidence available (S3 guideline) and subsequent discussion, the initially dissenting guideline members agreed with the new recommendation
9. Reconstructive procedures performed as part of more complex reconstructive procedures, e.g., using alloplastic (TEP) or autologous procedures (e.g., CCG) to replace the temporomandibular joint, should be performed in a single-stage procedure, if a combination with orthognathic surgical realignment is required.	EC	B	Criterion 1: K1.3 Not approved by majority (43%)		Not adaptated due to missing comments/discussion and insuffi-cient evidence

## 4. Discussion

Active condylar resorption will be completely asymptomatic in a majority of cases [6]. Only as few as approximately 25% of patients develop symptoms such as pain or functional limitations [5]. However, the severity of pain does not necessarily correlate with the extent of resorption [5].

In the stable phase, good functionality of the joint can usually be achieved in the absence of pain; the leading complaint here is deformity of the facial skeleton, often accompanied by deficient occlusion [1,9,10,11,18,19,23].

The therapy aims, therefore, are prevention of disease progression [11]; remediation of pain and of functional discomfort, e.g., in mastication and speech [9,10,11]; improvement of mandibular mobility and function [1,9,11]; restoration of proper static and dynamic occlusion and articulation, e.g., in class II malocclusion with/without anterior open bite [7]; correction or improvement of facial deformities and associated functional and aesthetic impairments, e.g., in mandibular retrognathia [1,11]; if necessary, the elimination of secondary sleep apnoea, which may occur due to airway obstruction in the advanced stage of ICR [1]; and rehabilitation of mandibular growth in adolescents, whose bone development is not yet complete [6].

Variability of symptoms makes clinical diagnosis difficult [24], which explains why imaging diagnostics (X-ray) need to be included among the mandatory examinations in addition to a clinical examination and palpation [25].

Idiopathic condylar resorption (ICR) is characterized by an altered shape (flattening, erosion) and decreased volume of the condyle and a 6–10% reduction in ramus height in conventional imaging [2,8,25,26,27]. However, when evaluating these diagnostic findings of conventional imaging, it is important to consider the underlying aetiology and concomitant clinical findings. For example, orthognathic transposition surgery, i.e., in mandibular advancement and/or set-back surgery, will inevitably induce remodelling, generating volume changes similar to those of condylar resorption [12,14,18,25]. Cephalometric lateral radiographs show characteristic features as in class II malocclusion with or without an anterior open bite, mandibular retrusion, low posterior facial height, a wider mandibular plane angle, narrowing of the oropharyngeal airway and a loss of ramus height [10,11,12,25]. In this context, however, it needs to be stressed that these radiological changes are typically found in ICR, but by themselves do not prove the diagnosis of ICR. Other factors, therefore, must be included to establish a definitive diagnosis to differentiate from degenerative diseases. For example, condylar resorption on conventional imaging is described as being characterized by altered shape (flattening, erosion) and decreased volume of the condyle, as well as a reduction of 6–10% in ramus height [2,8,13,18,26,27,28,29]. However, it is important to consider the underlying pathogenesis and concomitant clinic when evaluating these diagnostic findings of conventional imaging. For example, remodeling with expectable volume changes similar to those of condylar resorption inevitably occurs even with orthognathic surgery [13,14,18,28].

Direct comparison between patients who develop ICR postoperatively (after orthognathic surgery) and those who do not, however, reveals some significant differences. These concern the preoperative mandibular plane angle, which is significantly greater in patients who develop ICR, whereas the preoperative SNB angle, the overbite, posterior facial height and the ratio of posterior facial height to anterior facial height were significantly smaller [25].

In this context, it should be noted that, according to the literature, resorption after orthognathic surgery manifests in conventional two-dimensional imaging at the earliest 6 months after and at the latest 2 years after surgery [5,19].

For further diagnosis and treatment planning or exclusion, both the OMFS and the interdisciplinary guideline groups unanimously established a strong recommendation (grade A; “shall”) in favour of the use of three-dimensional imaging in spite of the low level of evidence available in the literature (LoE 4/k++; grade A).

Changes observed in advanced imaging and which may be associated with condylar resorption include osteophytes, disc dislocation, disc perforation and/or disc degradation, synovial hyperplasia, synovitis and loss of fibrocartilage [5]. Such advanced imaging methods include CT or CBCT [8,26,27,30], MRI [1,11,23] and nuclear–medical methods (scintigraphy) [10,23].

A controversial topic identified during the consensus process is the method of choice for three-dimensional imaging in ICR. According to the international literature, both CT and CBCT are suitable for diagnosing the initial presentation and the progression of ICR [23,30]. Due to its higher soft-tissue contrast resolution compared to CBCT, CT is considered to be particularly suitable for patients in whom the exclusion of other differential diagnoses is especially important [31,32].

According to Valladares-Neto et al., the pathognomonic loss of the cortical layer of the condyle, which is typically found in the erosion stage of idiopathic condylar resorption, can be assessed by both CT and MRI [5]. In addition, according to Valladares-Neto et al., CBCT imaging may be able to show the localization and allow for quantification of previously unidentified cases of idiopathic condylar resorption [5]. However, for the purpose of representation of specific bony structures, according to the international literature, it is CT which currently represents the imaging method of choice [5,33] (contrary to the recent German consensus-based S2k guideline “Dentale Digitale Volumentomographie” 2022, a fact which was discussed in depth during the guideline process), especially since CT, according to Cevidanes et al., has the best positive predictive value (84%) for the diagnosis of (osteo-) arthritis of the temporomandibular joint [30]. In assessing these conflicting statements, it should be noted that the German S2k guideline primarily reflects the current state of knowledge about CBCT regarding technical principles, its field of application and the associated doses of irradiation. Thus, while the above-mentioned international literature specifically covers the diagnosis of ICR, the German CBCT guideline addresses the definition of framework conditions in the application of CBCT within the entire field of dentistry and oral and maxillofacial surgery in Germany, and does not specifically focus on the assessment of ICR.

Against this background, we adapted our previous recommendations due to the good evidence available in the literature, and after consultation with the most competent scientific association with regard to questions concerning radiological imaging, i.e., the Deutsche Röntgengesellschaft e.V. (German Association of Radiology), which complied with our request for expert assessment and also became a member of the interdisciplinary guideline group. According to the consensus achieved, three-dimensional imaging (CT/CBCT) is the current diagnostic standard for imaging and the documentation of the extent of ICR, and for ruling out other differential diagnoses at initial presentation. Therefore, it should be used to document initial presentation and disease progression (LoE 4/k++, grade B). Equally, in cases of ICR, for specific questions regarding bony structures, CT or CBCT, which are basically equivalent for this indication, should be used as diagnostic tools (LoE 4/k++, B). Pathognomonic MRI findings of idiopathic condylar resorption include decreased condylar volume; anterior disc displacement with/without reduction of mouth opening; rarefaction or even loss of cortical continuity at the condylar surface; and thickened soft tissue with amorphous appearance occupying the intraarticular space between condyle and fossa [6,11]. The soft tissue with amorphous appearance is described as consisting of hyperplastic synovial tissue with little vascular component and usually no inflammation, overlying the condyle [11]. With regard to diagnosis of idiopathic condylar resorption, MRI offers advantages over CT, as it is more suitable for the assessment of both the anatomy and the position of the disc in MRI [5]. To date, however, there are no clinical studies on the value of diagnostic MRI imaging in idiopathic condylar resorption. This is why the guideline group decided to vote for an open recommendation after thorough discussion. Accordingly, an MRI examination may provide important additional information for the choice of surgical treatment and for the clarification of differential diagnoses, first and foremost for the purpose of the evaluation of soft tissue, especially of the disc (LoE 4/k++, 0).

Another advanced imaging technique is scintigraphy. The potential additional benefit of scintigraphy for differentiation between the active and stable states of resorption has been critically assessed by some authors [1,5,6,34]. However, due to the occurrence of both false-positive and false-negative results, the guideline group decided to issue an open recommendation in this case as well: scintigraphy may be indicated to determine the activity status of the resorption (active vs. stable), especially prior to surgical correction of occlusion and/or deformations (LoE 4/k+, 0).

If secondary condylar resorption is suspected, i.e., with a disease potentially causative for the resorption, the aetiology should be clarified by appropriate advanced diagnostics (LoE 4/k++, B).

For example, the presence of inflammation, condylar erosion, and/or cartilage damage may suggest an autoimmune cause for resorption [6]. Until now, serology has been considered the standard method for differential diagnosis of rheumatic diseases in condylar resorption. However, according to the relevant recommendations of a recently published consensus and evidence-based S3 guideline on inflammatory diseases of the temporomandibular joint (cf. S3 guideline “Inflammatory diseases of the temporomandibular joint: Juvenile idiopathic arthritis (JIA) and rheumatoid arthritis (RA) of the temporomandibular joint”, register number 007/061), the previous recommendation on serological diagnostics was rediscussed and reevaluated within the guideline group, based on this newly available higher level of evidence [21]. After detailed research, the recommendation developed is to primarily perform contrast-enhanced MR diagnostics of the temporomandibular joint in order to substantiate or rule out an autoimmune or rheumatoid disease as causative, and to perform serological diagnostics only if results are unclear (LoE 4/k+, B).

If secondary condylar resorption is evident, treatment of the underlying disease should be pursued in the first place (LoE 5/k+, B). If there is no underlying disease accountable for the resorption, there is also the option of starting with conservative therapy primarily. In addition to controlling symptoms, conservative procedures also may help decelerate the progression of ICR [1,7]. In a recent study from Zhou et al., it was shown that therapy with stabilisation splints can reduce excessive mechanical load on the TMJ, slow down bone destruction and promote condylar modelling [35].

Conservative therapy measures include functional therapy (e.g., occlusal splints) [35,36], accompanying orthodontic treatment [26], physiotherapy/manual therapy [10] and pharmacotherapy [1,10,33,37]. In the case of symptomatic active condylar resorption (pain, functional complaints), initially, attempts should be made to contain the symptoms with such conservative therapy procedures (LoE 4/k+, B). In particular, the traditional options of orthodontics (such as removable and fixed appliances and tooth extractions) can be applied in preparation for surgical therapy (LoE 4/k+, 0).

Recently, advances in pharmacotherapy have shown promising alternative interventions for deceleration of the progression of the disease, i.e., in reducing the extent of resorption. Whereas several studies have already indeed demonstrated good results of pharmacotherapy of rheumatoid arthritis of the temporomandibular joint [38,39,40], to date, no studies with higher levels of evidence exist focussing explicitly on pharmacotherapy of idiopathic condylar resorption. Existing studies focussing, e.g., on NSARs (2012) and antioxidants, omega-3-fatty acids, cytokine inhibitors and tetracyclines (2009) are either based on single case reports [7] or review articles, with inconsistent results [41]. The guideline group refrained from establishing specific recommendations regarding possible pharmacotherapy for idiopathic condylar resorption due to the still very limited evidence on this topic. It remains to be evaluated which medication will be successful in the long term in the treatment of condylar resorption, and what overall role medication will play in the treatment of ICR, especially considering the sometimes serious side effects, possible interactions and contraindications [1,10,33,37].

Surgical treatment may be indicated in the case of pronounced pain and massive functional disorders as well as for more serious deformities (LoE 4/k+, 0). However, timing and choice of surgical intervention remain controversial [1,9,10,18]. According to the available evidence and expert opinion, surgical treatment should generally be avoided in the active (progressive) phase of condylar resorption (LoE 4/k+, B). After the progressive phase subsides and transitions into the stable phase, if possible, stable occlusion should be (re)established in the long term (LoE 4/k+, B). Deformities of the facial skeleton caused by resorption can be corrected surgically in the stable phase, using the surgical procedures available for this purpose (LoE 4/k+, 0). In summary, the recommendation was established that if it is not possible to sufficiently control the symptoms of active condylar resorption (pain, functional limitations) by (otherwise efficient) conservative measures, condylectomy with subsequent reconstruction, e.g., by means of a costochondral graft (CCG) or comparable autologous procedures, or by microsurgical grafts or total alloplastic joint replacement (cf. S3 Guideline No. 007/106 “Total alloplastic temporomandibular joint replacement”, status 04/2020), if necessary in combination with orthognathic surgery, may be indicated (LoE 4/k+, 0).

Overall, the evaluation of surgical procedures for idiopathic condylar resorption is only possible with significant reservations due to the small number of cases in the available studies.

Basically, the following surgical procedures are available for the treatment of idiopathic condylar resorption: arthrocentesis [23]; arthroplasty in terms of discopexy [6,23] or discectomy [23]; arthroscopic condylectomy [10,19,42,43,44]; gap osteotomy [29]; partial autogenous TMJ reconstruction, e.g., by costochondral grafts (CCG) [10,19,43,44]; total alloplastic TMJ reconstruction [6,9,23,37,45]; and orthognathic surgery, e.g., BSSO, Le Fort I osteotomy, distraction osteogenesis and genioplasty (to establish stable occlusion and to correct accompanying deformities) [1,6,9,10,14,23,36]. However, due to the poor evidence base, some issues regarding surgical therapy remained to be addressed. For example, the lateral pterygoid muscle is known to play an important role in the functional movement of the mandible and should originally be attached at the condylar head level. Progressive stages of resorption in ICR, therefore, inevitably will alter the muscle’s functionality. With regard to this clinically important question, we put a special focus on the lateral pterygoid muscle during our literature search. Unfortunately, we found that—at least so far—there is hardly any specific literature concerning ICR dealing with the lateral pterygoid muscle such as [46], and none are MRI-related papers. Therefore, each individual case of ICR requires a specially tailored surgical treatment concept, taking into account the extent of resorption, occlusion, function and clinical symptoms.

Another highly controversial issue identified during the consensus process is discopexy, which—according to some authors—should be performed in conjunction with resection of hyperplastic synovia, if necessary [6,11]. In this context, the following rationale is given for discopexy: If active condylar resorption is detected at an early stage (anamnesis of disc displacement < 5 years), so that the displaced disc and condyle (residual volume at least 75%) can still be preserved, some authors recommend arthroplasty with discectomy [6,11]. The main idea is discopexy is expected to stop the progression of resorption. If necessary, hyperplastic synovia is resected, which is present mostly in the bilaminary zone. This procedure was controversial in discussions during the guideline development. While the OMFS working group supported the procedure proposed by Wolford et al. (discopexy at an early stage of ICR) by a majority ratio of 2/5 after two rounds of voting, the procedure was not endorsed by the mandate holders in the interdisciplinary Delphi process. Therefore, no recommendation could be established by the interdisciplinary guideline group regarding discopexy as proposed by Wolford et al. In the present guideline, however, there was a strong consensus that any arthroplastic procedures, e.g., for disc repositioning, condylar shave or similar, if required in combination with orthognathic surgery, should, as a rule, be performed as a two-stage procedure, i.e., the arthroplasty procedure should be performed first, followed by orthognathic surgery in a second step (LoE EC, B).

Additional new recommendations were established concerning the topic of temporomandibular joint replacement, both autologous and allogenic. Basically, both procedures can be considered.

Nevertheless, if adequate conservative and surgical measures with autologous reconstruction fail, or if multiple surgeries have already been performed in the region, the indication for arthroplasty with total alloplastic joint replacement should be considered, if symptoms are sufficiently severe (LoE 4/k+, B). In particular, if condylar resorption was caused by an inflammatory temporomandibular joint disease of the rheumatic type, this is seen as an indication for total alloplastic joint replacement [9,34,47,48]. Other than autologous reconstruction, alloplastic reconstruction may interrupt the autoimmune processes directed against the joint’s soft-tissue structures [47].

These recommendations regarding total alloplastic temporomandibular joint replacement were updated based on newly available studies with higher levels of evidence and supported by the respective recommendations of two recently published S3 guidelines (cf. S3 guideline “total alloplastic temporomandibular joint replacement”, register number 007/106; cf. S3 guideline “Inflammatory diseases of the temporomandibular joint: Juvenile idiopathic arthritis (JIA) and rheumatoid arthritis (RA) of the temporomandibular joint”, register number 007/061).

In individual cases, allogenic or autologous reconstructions of the condylar process and, if necessary, parts of the skull base, are combined with orthognathic surgery [6,10,42,45,49]. If so, the guideline group recommends that if performed as part of a more complex reconstructive procedure, e.g., using alloplastic or autologous procedures (e.g., CCG), if possible, replacement of the temporomandibular joint should be performed in a single-stage procedure if a combination with orthognathic surgery is required (LoE EC, B). In this context, it should be noted that isolated orthognathic surgery (without prior arthroplasty) is associated with an increased risk of recurrence of malocclusion and deformities due to further advancing or retriggering of resorption, especially during or 6–12 months after active resorption [1,9,10,50]. Therefore, orthognathic surgery without arthroplasty should be performed not earlier than 6 months after the end of active resorption (LoE 4/k+, B).

In general, the objective of any surgical therapy for condylar resorption is to minimize further compressive loads on the condyle [1,6,9], as compression of the condyle due to mechanical overload/misload (possibly triggering avascular necrosis) is considered to be one of the main risk factors for idiopathic condylar resorption [51,52]. Other risk factors are female gender aged 10–30 years [1,6,8,9,10,11,19,25,33], a wide angle between the occlusal and mandibular plane [1,6,11], nutritional deficiencies (e.g., in vitamin D and omega-3-fatty acids) [5,41,53], preexisting degenerative joint disease [3,30], genetic predisposition (polymorphisms of MMPs, vitamin D receptors and aromatase and oestrogen receptors) [5,41], a reduced capacity of the joint region to remodel, e.g., due to advanced age [9,15], systemic diseases (autoimmune, endocrine and metabolic) [1,6,9,12,54] and/or an inhibitory effect of low concentrations of certain sex hormones (especially oestrogens) [1,6,9,11,12,16,51,55].

In this context, another highly controversial issue necessitating thorough discussion among the members of the interdisciplinary guideline group was whether overloading/misloading of the temporomandibular joint may be caused by orthodontic treatment or orthognathic surgery, and thus whether orthodontic treatment or orthognathic surgery are among the risk factors for ICR. The incidence of condylar resorption after orthognathic surgery is reported in the literature to range from 1 to 31% [5,13,25]. However, some studies indicate that the high incidence reported may probably be due to measurement errors [5,6,18]. According to the international literature (low level of evidence, LoE 4), overloading/misloading can be caused by, e.g., occlusal misloading, possibly also in the context of specific therapy measures, e.g., orthodontic surgery [1,6,11,12,23,25,33]. Regarding orthodontic therapy as a possible risk factor, there is no clear evidence pointing either way in the literature. Orthodontics is often included as a regular therapy component in orthodontic–OMFS–surgical therapy protocols [11]. Since specific orthodontic measures can cause stress/compression on the joint [11,56], the literature points out that in the case of exclusive or concomitant orthodontic treatment, force vectors that cause such stress on the joint, e.g., through intermaxillary elastics, should be avoided in certain specific cases [1,56]. According to a recent meta-analysis by Francisco et al., it cannot be determined conclusively yet whether condylar resorptions indeed occur as a consequence of orthognathic surgery or if this is merely a coincidence due to the multifactorial aetiology [14]. Therefore, the guideline group refrained from a concrete recommendation regarding orthodontic treatment and orthognathic surgery as a possible risk factor.

A limitation of the study was the low level of evidence of most available published studies on ICR, as became again particularly noticeable during the literature research. The level of evidence was determined according to the AWMF rules and standards and the Oxford Criteria of 2011. This represents a major limitation, which the guideline group had to consider when establishing recommendations. As a result, some recommendations could not be assigned a strong grade of recommendation (A), as this would conflict with the low level of evidence available. In part, this meant that unequivocal statements were not always possible.

Another main limitation of this study relates to the Delphi process. As already analysed by Schmidt et al., this method, while offering the advantage of great flexibility to the members of the guideline group, is very time-consuming [21]. At the same time, the Delphi process also offers the additional great advantage of the anonymity with which voting results and comments can be evaluated. Thus, focus is drawn to factual content, more controversial approaches will be introduced and potential systematic bias as an effect of social interaction can be avoided. A potential approach for future guideline projects could be to complement an initial anonymous Delphi round, in which each voting member will initially be able to form his or her own opinion independently and represent it, thus avoiding peer pressure as a factor, by a subsequent constructive discussion round led by the guideline coordinator in the form of a formalized and structured consensus conference based on AWMF rules and standards (https://www.awmf.org/regelwerk/; accessed on 1 September 2022) to further clarify critical topics. This could save both time and resources without compromising the outcome.

Concerning the consensus process, the required number of rounds in the interdisciplinary phase K2 of the Delphi process was significantly higher than in the initial OMFS-exclusive Phase K1. This was probably caused by the heterogeneous composition of the interdisciplinary guideline group involving representatives from dental societies and nonsurgical societies.

## 5. Conclusions

In summary, the Delphi procedure provided for a profound, evidence-based, interdisciplinary exchange of ideas allowing for the development of the best possible expertise in the diagnosis and treatment of ICR. A review of the existing literature revealed that while pathognomonic features of ICR can often be observed on cephalometric lateral radiographs, a large proportion of these cases are asymptomatic (indeed, up to 75% are asymptomatic). Therefore, in ICR, it is of particular relevance to tailor therapy aims first and foremost to the patient’s symptoms and not primarily based on diagnostic imaging. It could also be established that three-dimensional imaging (CT/CBCT) is the current diagnostic standard for imaging and documentation of the extent of ICR. With regard to the surgical treatment of ICR, the issue of the small number of cases in the available studies persists, making evidence-based statements difficult. In the field of pharmacotherapy, current new therapeutic approaches are emerging, with long-term results and larger case series remaining to be evaluated. Furthermore, the guideline group discussed the procedure of discopexy in patients with ICR. Due to lack of studies of higher levels of evidence, no consensus could be reached, and future study results remain to be evaluated. To date, the aetiology of ICR is still unknown. Possible risk factors have already been identified, including orthognathic surgery. Whether condylar resorptions are a consequence of orthognathic surgery or merely occur in their context by mere coincidence requires further research.

## Figures and Tables

**Table 1:** Level of evidence based on Oxford Criteria 2011.

LoE	Study Type
1	Systematic review of randomized controlled clinical trial (RCT).
2	Randomized controlled clinical trial (RCT).
3	Non-randomized controlled clinical trial or follow-up study.
4	Case series or case-control study.
5	Case study, nonsystematic secondary literature, expert opinion, studies other than in vivo studies of human subjects (e.g., animal experiment, cadaver study) or consensus paper.

**Table 2:** Assessment of methodological quality according to SIGN Checklists.

Symbol	Criteria
++	High quality, overwhelming majority of criteria fulfilled (>75%), no risk or low risk of bias.
+	Acceptable quality, majority of criteria fulfilled (50–75%), medium risk of bias.
−	Low quality, majority of criteria not fulfilled (<50%), considerable risk of bias.
0	Unacceptable, study rejected due to insufficient quality.

**Table 3:** Rating of clinical relevance.

Symbol	Criteria
k++	High clinical relevance, overwhelming majority of criteria fulfilled (>75%).
k+	Acceptable clinical relevance, majority of criteria fulfilled (50–75%).
k−	Low clinical relevance, majority of criteria not fulfilled (<50%).
k0	Study without clinical relevance, study removed.

**Table 4:** Classification of strength of consensus according to AWMF rules and standards.

Agreement	AWMF Definition
>95%	Strong consensus
95–76%	Consensus
75–50%	Approval by majority
<50%	No consensus

## Data Availability

No publicly archived datasets available; data can be obtained from the authors upon reasonable request.

## Supplementary Materials

The following supporting information can be downloaded at: https://www.mdpi.com/article/10.3390/jcm12154946/s1, Table S1. Members of the working group “oral and maxillofacial surgeons” (OMFS) of the German Association for Oral and Maxillofacial Surgery (Deutsche Gesellschaft für Mund-, Kiefer- und Gesichtschirurgie—DGMKG); Table S2. Participating medical Expert Associations including the elected Representatives; Table S3. Laboratory parameters for differential diagnosis of inflammatory temporomandibular joint diseases in form of rheumatoid arthritis; Table S4. Summary table of studies for which the validity of diagnosis ICR cannot be guaranteed or papers for which not only patients with ICR have been evaluated.
