# Supplementary material for: Controversial Aspects of Diagnostics and Therapy of Idiopathic Condylar Resorption: An Analysis of Evidence- and Consensus-Based Recommendations Based on an Interdisciplinary Guideline Project
Source: J Clin Med. 2023 Jul 27;12(15):4946. doi: 10.3390/jcm12154946 (PMC10419428; doi:10.3390/jcm12154946)
Supplement: Supplementary file 1 [file jcm-12-04946-s001.zip › jcm-2442100-supplementary.pdf]

[1] **Table S1.** Members of the working group "oral and maxillofacial surgeons" (OMFS) of the German Association for Oral and Maxillofacial Surgery (Deutsche Gesellschaft für Mund-, Kiefer- und Gesichtschirurgie—DGMKG).

Members	Department/Clinic/Medical Practice
Univ.-Prof. Dr. med. Dr. med. dent.<br>Johannes Kleinheinz	Klinik für Mund-, Kiefer und Gesichtschirurgie<br>Universitätsklinikum Münster<br>Albert-Schweitzer-Campus 1<br>48149 Münster
Univ.-Prof. Dr. med. Dr. med. dent.<br>Andreas Kolk	Klink für Mund-, Kiefer- und Gesichtschirurgie<br>Medizinische Universität Innsbruck<br>Christoph-Probst-Platz 1, Innrain 52 A<br>6020 Innsbruck<br>Österreich
Univ.-Prof. Dr. med. Dr. med. dent.,<br>Prof. h.c. (BNMU, Kyiv)<br>Andreas Neff	Klinik und Poliklinik für Mund-, Kiefer- und Gesichtschirurgie<br>Universitätsklinikum Marburg<br>Baldingerstraße<br>35043 Marburg
Prof. Dr. med. Dr. med. dent.<br>Christoph Pautke	Medizin & Ästhetik Praxisklinik für Mund-, Kiefer- und plastische Gesichtschirurgie<br>Lenbachplatz 2a<br>80333 München
Dr. med. Dr. med. dent.<br>Andreas Schön	Praxisklinik für Mund-, Kiefer- und Gesichtschirurgie<br>Bahnstraße 140<br>53842 Troisdorf
Dr. med. Dr. med. dent.<br>Marcus Teschke	Praxis für Gesichtschirurgie & Kiefergelenkschirurgie<br>Brockdorfstr.90<br>22149 Hamburg
Dr. med. Dr. med. dent.<br>Astrid Toferer	Klinische Abteilung für Mund-, Kiefer- und Gesichtschirurgie<br>Medizinische Universität Graz<br>Auenbruggerplatz 5/6<br>8036 Graz

Table S2. Participating medical Expert Associations including the elected Representatives.

Association	Elected Reprasentative
Deutsche Gesellschaft für Funktionsdiagnostik und –therapie(DGFDT)	Priv.-Doz. Dr. med. dent. M. Oliver Ahlers
Deutsche Gesellschaft für Kieferorthopädie (DGKFO)	Priv.-Doz. Dr. med. dent. Dr. sc. hum. Christian Kirschneck
Deutsche Gesellschaft für Kieferorthopädie (DGKFO)	Univ.-Prof. Dr. med. dent. Christopher J. Lux
Deutsche Gesellschaft für Prothetische Zahnmedizin und Biomaterialien e.V. (DGPro)	Univ.-Prof. Dr. med. dent. Peter Ottl
Deutsche Röntgengesellschaft e.V. (DRG)	Univ.-Prof. Dr. med. Gabriele Krombach
Deutscher Verband für Physiotherapie e.V. (ZVK)	Ima Feurer

Table S3. Laboratory parameters for differential diagnosis of inflammatory temporomandibular joint diseases in form of rheumatoid arthritis[1].

Blood sedimentation	frequently elevated in (untreated) rheumatoid arthritis

[1] Cf. S3 guideline „Management der frühen rheumatoiden Arthritis“ 2019, register number 060 - 002

	non-specific (also in anemia, other inflammatory diseases)
C-reactive protein (CRP)	quantitatively more accurate and shows a quicker response than blood sedimentation, better identification, detection of so-called acute phase reaction (disease activity), equally non-specific as blood sedimentation
Blood count	in case of prolonged active disease: inflammatory anemia (normochromic or hypochromic, normocytic), thrombocytosis
IgM rheumatoid factor (RF)	positive in 65-80% of patients with rheumatoid arthritis; specificity approx. 80%, as also detectable in collagenosis, viral hepatitis, malignancy and (rarely) also in healthy individuals
Antibodies against citrullinated proteins/peptides (ACPA)	highly specific for rheumatoid arthritis (> 95%) and as sensitive (64-86%) as rheumatoid factor; can be positive even before clinical manifestation of rheumatoid arthritis and is highly predictive of a chronic course and erosive course in presence of early arthritis
Urine analysis	exclusion of hematuria, proteinuria as an indication of other diseases (e.g., collagenoses)
Antinuclear antibodies (ANA)	differential diagnostic indication for collagenoses (e.g. systemic lupus erythematosus), weakly positive also in rheumatoid arthritis or healthy individuals
Antineutrophil Cytoplasmic Antibody (ANCA)	differential diagnostic indication for vasculitis (e.g. granulomatosis with polyangiitis)
HLA-B27	differential diagnostic indication for spondylarthritis
Uric acid / joint punctate	differentiation from polyarticular gout (rare) and infectious arthritis (monarthrotic, large joints)

Source 1: Cf. S3 guideline „Management der frühen rheumatoiden Arthritis“ 2019, register number 060 – 002“, https://register.awmf.org/de/leitlinien/detail/060-002, last downloaded 04.07.2023.

Table S4. Summary table of studies for which the validity of diagnosis ICR cannot be guaranteed or papers for which not only patients with ICR have been evaluated.

author, year, LoE	Risk of confusion with degenerative joint diseases
(Alsabban et al. 2018, 4/k+)	Epidemiological survey among craniomaxillofacial surgeons, thereby no concrete specification of diagnostic criteria of ICR
(Nicolielo et al. 2017, 5/k++)	Summary article, which also included articles on the effects of oestrogen on TMJ osteoarthritis as it is hypothesized that similar hormonal receptor effects and disease mechanisms may play a role
(Posnick und Fantuzzo 2007, 4/k+)	Case series, one case with progressive condylar resorption in context of JIA, one case with idiopathic condylar resorption
(Qiu et al. 2010, 4/k+)	Study evaluating endoscopically assisted reconstruction of the mandibular condyle with a costochondral graft through a modified preauricular approach, patients with diagnoses including osteoarthrosis, ankylosis, tumours, idiopathic condylar resorption, comminuted condylar fracture, and chronic osteomyelitis of the temporomandibular joint were evaluated

(Troulis und Kaban 2001, 4/k+)	Study evaluating endoscopic approach to the ramus/condyle unit, patients with diagnoses of idiopathic condylar resorption, subcondylar fracture, mandibular prognathism, condylar hyperplasia, and mandibular asymmetry
(Troulis et al. 2004, 4/k+)	Study evaluating endoscopic condylectomy, both patients with ICR and degenerative joint disease, and malunion of a fractured condyle were evaluated
(Valladares-Neto et al. 2014, 5/k++)	This study reviewed the response of the TMJ to mandibular advancement surgery by analyzing certain risk factors, which included three TMJ changes (disk displacement, arthralgia, and condylar resorption) and two treatment variables (fixation techniques and the amount of advancement). Therefore, a precise separation between cause and consequence is not always possible.
